# Effect of receptive provocation as a therapeutic approach for cognitive and consciousness improvement among traumatic brain injury patients in India

**DOI:** 10.6026/9732063002001979

**Published:** 2024-12-31

**Authors:** Sudha Devadoss, Theranirajan Ethiraj, Shankar Shanmugam Rajendran, Anandhi Duraikannu, Shabana Nisarahamed, Deepika Ramalingam, Sundari Mani

**Affiliations:** 1 Department of Medical Surgical Nursing, College of Nursing, Madras Medical College, The TN Dr MGR Medical University, Chennai, Tamil Nadu, India; 2 Department of Pediatrics, Madras Medical College, The TN Dr MGR Medical University, Chennai, Tamil Nadu, India; 3 Department of Pediatrics Nursing, College of Nursing, Madras Medical College, The TN Dr MGR Medical University, Chennai, Tamil Nadu, India; 4 Department of Medical Surgical Nursing, College of Nursing, Madras Medical College, The TN Dr MGR Medical University, Chennai, Tamil Nadu, India

**Keywords:** Traumatic brain injury, cognitive function, level of consciousness, receptive provocation therapy, multisensory stimulation

## Abstract

Traumatic brain injury (TBI) is a global health concern, often resulting in cognitive deficits and altered states of consciousness. This study evaluated the effectiveness of Receptive Provocation (RP) therapy in improving mental function and consciousness levels among TBI patients. 70 patients were randomly assigned to experimental and control groups, with the experimental group undergoing RP therapy for 20-25 minutes daily over seven days, while the control group received routine care. Post-test assessments on day 8 revealed significant improvements in the experimental group's cognitive function and consciousness levels (p=0.001), whereas no significant changes were observed in the control group. These findings suggest that RP therapy is an effective intervention for enhancing cognitive and consciousness outcomes in TBI patients.

## Background:

Traumatic brain injury (TBI) is the term for disruption in brain function brought on by a traumatic event, such as a blow to the head or a deep cut [1]. Traumatic brain injury can lead to physical, cognitive, emotional and behavioral manifestations [2]. Some types of TBI can cause temporary or short-term difficulty with normal brain function, including problems with individual thinking, understanding, movements, communication & activities [3]. Severe traumatic brain injury results in high morbidity and mortality, mainly when young adults are concerned and are known to be a group at significant risk of TBI [4]. An estimated 1.5 to 2 million people in India sustain a yearly TBI. Road traffic accidents are an essential cause, contributing to about 60% of all TBIs. Falls and violence are other significant causes, accounting for about 20% and 10% of TBIs, respectively [5, 6]. This represents more than (611) TBI-related hospitalizations and (190) TBI-related deaths per day in India in 2021. Improvement in the level of consciousness is considered an indicator of recovery from traumatic brain injury. Receptive provocation is a simple, non-invasive intervention with a potentially positive effect on LOC [10, 11, 12, 13- 14]. Therefore, it is of interest to evaluate the impact of receptive provocation on cognitive function and LOC among patients hospitalized in the neuro-intensive care unit.

## Methodology:

## Statement of the problem:

A study to assess the effectiveness of receptive provocation on cognitive function and level of consciousness among traumatic brain injury patients at tertiary care center, Chennai.

## Objectives of the study:

The cognitive function and level of consciousness in patients with traumatic brain injury (TBI) before any intervention was evaluated. It will also investigate the effects of Receptive Provocation on these patients and compare their cognitive function and consciousness levels before and after the intervention. Additionally, the research will explore the relationship between post-assessment cognitive function and consciousness levels in relation to various demographic factors. The first hypothesis (H_1_) suggests that there will be a significant difference in post-test cognitive function and consciousness levels between the experimental group and the control group. The second hypothesis (H_2_) posits a notable association between the experimental group's post-test cognitive function and consciousness levels and their demographic characteristics.

## Materials and Methods:

Patients with traumatic head injuries who were admitted to the neurology department participated in a quantitative study employing a pre-test - post-test alone Randomized Controlled Trial methodology in Rajiv Gandhi Government General Hospital after obtaining permission from the Director and Head of the department. A pilot study was conducted among 10% of the calculated sample size to establish the feasibility. The investigator chose 70 patients using simple random and lottery sampling techniques. The participants were then divided into experimental and control groups. After acquiring demographic information, the experimental group (35 patients) underwent receptive provocation therapy, including sight, sound, touch, taste and smell stimuli to elicit responses in patients lasting (20-25) minutes for seven consecutive days, and the control group (35 patients) received routine care. On the 8th day, post-interventional cognitive function and level of consciousness were assessed. The collected data was tabulated and analyzed using appropriate Descriptive statistics (Frequency and %) and the difference between the pre-test and post-test was computed using the paired t-test and Mc Nemar's test. Hence, one-way ANOVA F-test and t-test was used to evaluate the correlation between the score level and demographic characteristics.

## Results:

The background characteristics of participants in both groups were aged between 21-30 years, (34.29%) experimental group and (28.57%) control group. All participants in both groups were male. The primary religion was Hindu, comprising (80.00%) experimental group and (71.43%) control group. Regarding residential status, more than half lived in urban areas, with (51.43%) experimental group and (57.14%) control group. The largest segment of both groups was unmarried, with most participants having a high school level of education; occupational status was skilled labor, accounting for (31.43% of) the experimental group and (34.29% of) the control group (Table 1). Income levels were mainly within the range of Rs. 5001 - Rs. 17000. The severity of traumatic brain injuries was primarily severe, and personal habits such as alcohol use were noted in nearly half of each group. The pretest level of cognitive function scores for the experimental and control groups are displayed. The experimental group scored (11.43%) and the control group (17.15%) higher on the Level I cognitive function scale. In both groups, most individuals (88.57% in the experimental group and 82.85% in the control group) scored at Level 2. With a p-value of 0.49 and a Chi-square test result of 0.47, there appears to be no statistically significant difference in the cognitive function scores between the two groups. It shows that most participants exhibited moderate levels of consciousness (91.43%) in the experimental group and (85.71%) in the control group. The Chi-square test resulted in a value of 0.56 and a p-value of 0.45, indicating no significant statistical differences between the groups regarding their pretest level of consciousness scores (Table 2). The majority of the experimental group had mild cognitive impairment, representing 62.86%, compared to 31.43% in the control group. Conversely, 37.14% of the experimental group and a significantly higher 68.57% of the control group had moderate cognitive function. The Chi-square test showed a significant difference between the groups, with a value of 6.94 and a p-value of 0.001 (Table 3).

A significant majority of the experimental group, 68.57%, displayed a mild level of consciousness, only 20.00% in the control group. Conversely, only 31.43% of the experimental group scored at a moderate level of consciousness, and in the control group at 80.00% (Table 4). It shows a mean difference of 0.86 (t=5.44, p=0.001, S), indicating a statistically significant improvement in cognitive function for the experimental group (Table 5). Table 6 outlines the pre-test and post-test coma scores, showing substantial improvements in the experimental group's eye-opening, verbal, and motor responses (all p=0.001). The result suggests that while the intervention greatly improved initial scores, subsequent measurements stabilized without significant change.

Table 7 assesses the association between post-test cognitive function scores and demographic variables, finding substantial associations related to the severity of traumatic brain injury and associated injuries within the experimental group (χ^2^ =7.74, p=0.05). In the Experiment group, significant associations were found in the severity of traumatic brain injury and associated injuries with cognitive function. Patients with severe injuries displayed better cognitive recovery (54.54% moderate function) than those with moderate or mild injuries. In the Control group, the result suggests that some demographic variables and clinical factors do not significantly impact cognitive outcomes in the control group. Table 8 explores the association between post-test levels of consciousness and demographic variables, revealing that patients with severe injuries demonstrated higher percentages of moderate alterations in consciousness. In the Experiment group, the result was that patients with severe injuries demonstrated a higher percentage of moderate alterations in consciousness (45.45%). Also, individuals with no associated injuries predominantly showed mild alterations (100.00%). The GCS scores showed a significant trend, with those scoring 6-7 having a more moderate alteration in consciousness (57.14%). The result suggests that some demographic variables and clinical factors do not significantly impact consciousness scores in the control group.

## Discussion:

The pretest level of cognitive function and consciousness in individuals with traumatic brain injuries levels between experimental and control groups were compared. For cognitive function, 88.57% of the experimental group and 82.85% of the control group scored at Level 2, while 11.43% of the experimental and 17.15% of the control were at Level 1, showing a statistically non-significant difference. Regarding consciousness levels, 91.43% of the experimental and 85.71% of the control group were categorized as moderate. In comparison, 8.57% of the experimental and 14.29% of the control was mild, indicating a non-significant statistic. The above findings are similar to a study conducted by Murtaugh *et al.* [7]; pretest consciousness levels were assessed using the Full Outline of UN Responsiveness (FOUR) score. They found that 90% of their experimental and 86% of the control groups scored moderately. These results support the comparability in baseline consciousness levels across different studies. The study's second objective is to determine Receptive Provocation's effectiveness on cognitive function and level of consciousness among traumatic brain injury patients. The study evaluates the efficacy of Receptive Provocation in improving cognitive function and consciousness levels among traumatic brain injury patients which showed that after the intervention, the experimental group showed a significant improvement in cognitive function scores, with a gain of 10.60%, increasing from a pretest mean of 66.00% to a posttest mean of 76.60%. Conversely, the control group, which received routine care, exhibited only a marginal gain of 1.70%. These findings show the benefits of Receptive Provocation in improving cognitive and consciousness outcomes compared to routine care. This was supported by a study conducted by Burman *et al.* [8] investigated a similar intervention termed "Cognitive Stimulation Therapy" in TBI patients. Their post-intervention results showed an improvement in cognitive function by 12%, slightly higher than this finding of 10.60%. This suggests that Receptive Provocation could be comparably effective in cognitive enhancement. The third objective of the study is to compare the pretest and post-test levels of cognitive function and level of consciousness among traumatic brain injury patients.

In the present study, the experimental group showed a significant mean increase in cognitive function scores from pretest (6.60 ± 0.69) to posttest (7.66 ± 0.48) with a mean difference of 1.06, demonstrating statistical significance (t=8.18, p=0.001). The control group exhibited a non-significant increase (mean difference 0.98; t=1.85, p=0.06). In coma scores, both groups significantly increased pretest scores for all categories (p=0.001). Hence, from the above findings, Hypothesis H1 was accepted. This was supported by a study conducted by Ahorsu *et al.* [9], who found significant improvements in cognitive function from pretest to posttest in their research using neuro-modulation techniques, with a mean increase of 1.02 points, which is comparable to the rise of 1.06 points. Their results support the potential for interventions to enhance cognitive recovery significantly. The fourth objective of the study is to associate the post-test level of mental function and level of consciousness among patients with the selected demographic variables. The present study findings revealed that the patients with mild injury severity, no associated injuries, and stable vital signs exhibited mild cognitive function scores. Similarly, those with mild injury severity, no related injuries, and higher initial levels of consciousness showed milder consciousness scores at the post-test. These relationships were statistically significant, as confirmed by chi-square tests, highlighting that less severe injury conditions are associated with better recovery outcomes. Hence, from the above findings, Hypothesis H_2_ was accepted. This was supported by a study conducted by Dell *et al.* [10], who examined demographic influences on post-test cognitive outcomes in TBI patients. Their findings that younger patients and those without comorbid conditions showed better recovery align with this observation that less severe injuries correlate with better outcomes.

## Conclusion:

Specialized interventions, such as Receptive Provocation (RP), are essential in the rehabilitation of traumatic brain injury (TBI) patients, significantly enhancing cognitive function and consciousness levels. Nurses play a pivotal role in successfully implementing these interventions by leveraging their expertise in administering and monitoring therapies, ensuring patient safety, and achieving optimal outcomes. The necessity of continuous professional development and training for nurses in advanced TBI care techniques to keep them abreast of innovative treatment strategies is emphasized. Furthermore, nurses are critical in educating patients and their families about TBI management, setting realistic expectations and promoting adherence to treatment plans, contributing to immediate recovery and long-term well-being. These findings underscore the broader importance of integrating specialized therapies like RP and enhancing the skills of nursing professionals to improve the quality of care for TBI patients.

## Figures and Tables

**Table 1:** Comparison of pretest level of cognitive function score

**Level of cognitive**	**Experimental group**		**Control group**		**Chi-square test**
**function score**	**n**	**%**	**n**	**%**	
Level 1	4	11.43%	6	17.15%	χ^2^=0.47
Level 2	31	88.57%	29	82.85%	p=0.49
Level 3	0	0.00%	0	0.00%	(NS) DF=1
Total	35	100%	35	100%	

**Table 2:** Comparison of pretest level of consciousness score

**Level of consciousness score**	**Experimental group**		**Control group**		**Chi-square test**
	**n**	**%**	**n**	**%**	
Mild	3	8.57%	5	14.29%	χ^2^=0.56
Moderate	32	91.43%	30	85.71%	p=0.45
Severe	0	0.00%	0	0.00%	(NS) DF=2
Total	35	100%	35	100%	

**Table 3:** Comparison of post-test level of cognitive function score

**Level of Cognitive function score**	**Experimental group**		**Control group**		**Chi-square test**
	**n**	**%**	**n**	**%**	
Mild	22	62.86%	11	31.43%	χ^2^=6.94
Moderate	13	37.14%	24	68.57%	p=0.001***
Severe	0	0.00%	0	0.00%	(S) DF=1
Total	35	100%	35	100%	

**Table 4:** Comparison of posttest level of consciousness score

**Level of consciousness score**	**Experimental group**		**Control group**		**Chi-square test**
	**n**	**%**	**n**	**%**	
Mild	24	68.57%	7	20.00%	χ^2^=16.73
Moderate	11	31.43%	28	80.00%	p=0.001***
Severe	0	0.00%	0	0.00%	(S) DF=1
Total	35	100%	35	100%	

**Table 5:** Experimental and control group cognitive function score

	**Group**				**Mean difference**	**Student independent t-test**
	**Experimental (n=35)**		**Control (n=35)**			
	**Mean**	**SD**	**Mean**	**SD**		
Pre-test	6.6	0.69	6.63	0.84	0.03	t=0.16 p=0.88(NS)
Post-test	7.66	0.48	6.8	0.8	0.86	t=5.44 p=0.001***(S)

**Table 6:** Pretest and posttest coma score

		**Group**				**Mean difference**	**Student paired t-test**
		**Experimental**		**Control**			
		**Mean**	**SD**	**Mean**	**SD**		
Pretest	Eye-opening Response	2.43	0.56	3.4	0.5	0.97	t=34.00 p=0.001***(S)
	Verbal Response	2.6	0.55	3.63	0.6	1.03	t=36.00 p=0.001***(S)
	Motor Response	2.6	0.55	3.69	0.53	1.09	t=22.61 p=0.001***(S)
	TOTAL	7.6	0.95	10.6	0.98	3	t=29.88 p=0.001***(S)

**Table 7:** Association between posttest level of cognitive function score and patients' demographic and clinical variables

**Demographic variables**		**Cognitive Function**					**Chi-square test**
		**Moderate**		**Mild**			
		**n**	**%**	**n**	**%**	n	
Severity of traumatic brain injury	Mild	0	0.00%	2	100%	2	χ^2^=7.74 p=0.05*(S)
	Moderate	1	9.09%	10	91.82%	11	
	Severe	12	54.54%	10	45.46%	22	
	No injuries	1	10.00%	9	90%	10	
Associated injuries	Limb fracture	3	27.27%	8	72.73%	11	χ^2^=8.05 p=0.05*(S)
	Rib fracture	5	62.50%	3	37.50%	8	
	Others	4	66.67%	2	33.33%	6	
Vital signs	Stable	2	18.33%	13	86.67%	15	χ^2^=6.37 p=0.01**(S)
	Unstable	11	55.00%	9	45.00%	20	

**Table 8:** Association between posttest level of consciousness score and patients' demographic variables

**Demographic variables**		**Cognitive Function**					**Chi-square test**
		**Moderate**		**Mild**			
		**n**	**%**	**n**	**%**	n	
Severity of traumatic brain injury	Mild	0	0.00%	2	100%	2	χ^2^=6.10 p=0.05*(S)
	Moderate	1	9.09%	10	91.82%	11	
	Severe	10	45.45%	12	45.46%	22	
	No injuries	0	0.00%	10	100%	10	
Associated injuries	Limb fracture	4	36.36%	7	63.64%	11	χ^2^=8.30 p=0.05*(S)
	Rib fracture	3	37.50%	5	62.50%	8	
	Others	4	66.67%	2	33.33%	6	
	GCS score 6-7	8	57.14%	6	43.86%	14	
Level of consciousness	GCS score 7-9	3	15.78%	16	84.22%	19	χ^2^=7.36 p=0.05**(S)
	GCS score 9-10	0	0.00%	2	100.00%	2	
